# ESR Essentials: gadolinium-wise MRI—practice recommendations by the European Society for Magnetic Resonance in Medicine and Biology

**DOI:** 10.1007/s00330-024-11214-4

**Published:** 2024-12-19

**Authors:** Carlo C. Quattrocchi, Àlex Rovira, Aart J. van der Molen, Carlo A. Mallio

**Affiliations:** 1 Centre for Medical Sciences—CISMed, University of Trento, Trento, Italy; 2 Azienda Provinciale per I Servizi Sanitari—APSS—Provincia Autonoma di Trento, Trento, Italy; 3 Section of Neuroradiology, Department of Radiology, Autonomous University of Barcelona and Hospital Vall d’Hebron, Barcelona, Spain; 4 Department of Radiology, Leiden University Medical Center, Leiden, The Netherlands; 5 Fondazione Policlinico Universitario Campus Bio-Medico, Roma, Italy; 6 Research Unit of Diagnostic Imaging and Interventional Radiology, Department of Medicine and Surgery, Università Campus Bio-Medico di Roma, Roma, Italy

**Keywords:** Magnetic resonance imaging, Contrast media, Patient safety, Gadolinium deposition, Clinical decision-making

## Abstract

**Abstract:**

The Gadolinium Research and Education Committee (GREC) is a working group of the European Society for Magnetic Resonance in Medicine and Biology (ESMRMB), established in 2016. The aim of the committee is to monitor scientific evidence for a continuous quality and safety improvement of enhanced MRI using gadolinium-based contrast agents (GBCAs), and also assess potential alternatives.

The scope of the present article is to describe the level of evidence concerning safety beyond the single patient (access to community and environmental impact), justification and optimization of the use of GBCAs beyond dosage (appropriateness and influence on clinical decision making), dose reduction with the use of AI (benefits and pitfalls), the advent of next-generation GBCAs (based on currently available data).

**Clinical relevance:**

GBCAs are extensively used in MRI and influence clinical decision-making. Their use to enhance the contrast-to-noise ratio is guided by recommendations from subspecialty societies. These guidelines advocate for GBCA use as an additional tool when necessary, ensuring they are administered at the lowest reasonable dose.

**Key Points:**

- *The choice of GBCAs used in radiology should be based on MRI cost-effectiveness, MRI access to the patient community, and impact on the environment, (evidence level: low).*
- *GBCA optimization includes reducing GBCA volume burden and increasing appropriateness by including post-contrast enhancement in MRI protocols, depending on clinical indications, (evidence level: moderate).*
- *Next-generation GBCAs show higher kinetic stability and higher T1 relaxivity when compared with standard macrocyclic GBCAs allowing comparable diagnostic accuracy at lower doses, (evidence level: moderate).*

## Key recommendations

- For safety procedures regarding the use of gadolinium-based contrast agents (GBCAs) refer to ESUR-CMSC guidelines. Thinking beyond single-patient safety, the choice of intravenous injection of GBCA in radiology should also incorporate MRI cost-effectiveness, MRI access to the patient community, and impact on the environment. Multi-society joint statements and cooperative actions are recommended to increase awareness of the consequences of inefficient use of GBCA (evidence level: low—expert opinions and recommendations exist; in vitro research is available, but further investigation is necessary).
- The use of GBCAs is justified only when appropriate or when it can potentially influence clinical decisions, based on the patient’s history and pre-MRI clinical assessment (evidence level: moderate—several recommendations exist, but further research is necessary to increase confidence).
- GBCA standard dose is 0.1 mmol/kg body weight for most applications of gadobutrol, gadoterate meglumine, and gadoteridol; 0.05 mmol/kg body weight for liver imaging using gadobenate dimeglumine, and 0.025 mmol/kg body weight for liver imaging using gadoxetate disodium. Dose optimization is recommended, as administering a lower-than-standard dose can still provide equally effective diagnostic information. Virtual contrast-enhancement with zero-dose is unlikely to reflect pharmacokinetic events (evidence level: moderate—further research is necessary and lowering the dose can still affect diagnostic yield under specific circumstances).
- Next-generation GBCAs have been developed to show higher kinetic stability and higher T1 relaxivity when compared with standard macrocyclic GBCAs. The additional benefit of these features is that comparable diagnostic efficacy is obtained with a lower (0.05 mmol/kg for gadopiclenol and 0.04 mmol/kg for gadoquatrane) than the current standard dose (evidence level: moderate—results of controlled phase III clinical trials are available).

## Introduction

Since their introduction in 1988 more than 800 million GBCA doses have been administered worldwide, with an estimated 63 million doses in 2023. Most GBCAs are used for brain, cardiovascular, abdominal, and musculoskeletal MRI examinations. According to World Health Organization reports, 20–40% of all health resources might be wasted as a consequence of operational inefficiency [1]. Allocative efficiency (related to the appropriate use of GBCA in MRI) and technical efficiency (related to the type and dose of GBCA administered to patients) must be maximized, such that the highest diagnostic value is obtained with the lowest access to GBCA-related resources. Therefore, the wise use and choice of GBCA must be promoted with recommendations based on evidence-based medicine.

The Gadolinium Research and Education Committee (GREC) is a working group of the European Society for Magnetic Resonance in Medicine and Biology (ESMRMB), established in 2016. The committee is composed of global academic experts who share multidisciplinary competencies to deal with complex issues at a cultural bridge across radiology, chemistry, physics, biology, environmental science, and informatics. The aim of the committee is to monitor scientific evidence for a continuous quality and safety improvement of enhanced MRI using GBCAs and assess potential alternatives [2, 3].

The scope of the present article is to summarize what the radiologist should know in terms of safety beyond the current guidelines, justification of use, dosing, the role of AI, and the availability of new GBCAs for clinical use.

## Safety beyond the patient: increasing accessibility to MRI and environmental sustainability

Regarding safety procedures before and after single-patient gadolinium-enhanced MRI scans, the Contrast Media Safety Committee of the European Society of Urogenital Radiology (ESUR-CMSC) guidelines are the current standard of reference and are periodically reviewed and updated [4]. Thinking beyond the safety of the single patient, the choice of intravenous injection of GBCA in radiology should also incorporate MRI cost-effectiveness, also considering MRI access to the patient community [5]. Based on the multiparametric nature of MRI, there has been an open debate on the additional diagnostic benefit provided by contrast-enhanced images [6, 7]. Pre-exam patient work-up, the risk of extravasation, allergy-like or chemotoxic reactions, the risk of gadolinium retention into tissues despite the lack of clinical consequences [8, 9], the need for additional staff (i.e., nurses, anesthesiologists), longer times for imaging protocols and post-scan safety checks are the main factors that increase the complexity and the patient discomfort when full MRI scan protocols that include GBCA injection are prescribed. In addition, the systematic inclusion of GBCA in MRI protocols reduces the number of MRI scans per h/per day, therefore decreasing accessibility. All these considerations must be taken into account and cost-effectiveness analyses of unenhanced (in some contexts also called abbreviated protocols) vs enhanced full MRI protocols should be promoted in different clinical applications [10–13].

The other major safety concern regards the potential environmental impact of anthropogenic gadolinium emissions [14]. The seven Rs (Rethink, Refuse excess, Reduce volumes and waste, Reuse and recycle, Reintegrate, Responsibility, and Remember) also apply to GBCAs [15]. In fact, 19 tons of gadolinium are annually emitted in Europe (for comparison 21 tons are estimated in the USA) and a two-fold increase in annual anthropogenic gadolinium has been observed in the last 15 years [16] (for comparison 7.7 fold increase of gadolinium has been reported between 1996 and 2020 in river water in Japan) [17]. The annual quantity of gadolinium per MRI scanner has remained unchanged (estimated at 2.7 kg per MRI in 2018) and the number of MRI scanners has increased [18]. Together, Germany, France, and Italy contribute 40% of Europe’s annual gadolinium flux. Of these, estimated on seaboards, 43% of anthropogenic gadolinium is emitted to the Atlantic Ocean, 24% to the Black Sea, 23% to the Mediterranean Sea, and 9% to the Baltic Sea [19]. Strategies to reduce the environmental impact of gadolinium include adherence to recovery circuits of unused GBCA (collection and recycling contrast media leftovers found at the bottom of used bottles), as well as using urine bags for home or urination in dedicated bathrooms at the hospital to prevent/mitigate emission in sewage water [14, 15].

It is not easy to estimate the environmental impact of one specific agent among the several factors affecting biological systems in real-life contexts. Nevertheless, in vitro experiments show that biomarkers of metabolic capacity and oxidative stress are influenced by the accumulation of gadolinium in biology models such as clams [20–22] or plants [23]. Recently, the effect of increasing levels of exposure to gadolinium has shown teratogenic effects on freshwater cnidarian *Hydra* *vulgaris* [24].

Last but not least, the progressive increase in demand for gadolinium must not be ignored. An enormous amount has been naturally extracted so far, with China being the dominant supplier globally, and sustainability balance means consideration of environmental consequences of the entire supply chain process [25].

In summary, multi-society joint statements and cooperative actions are recommended to increase medical community and population awareness of the consequences of inefficient use of GBCA.

## Optimization beyond dosage: appropriateness and clinical indications

**Fig. 1:**
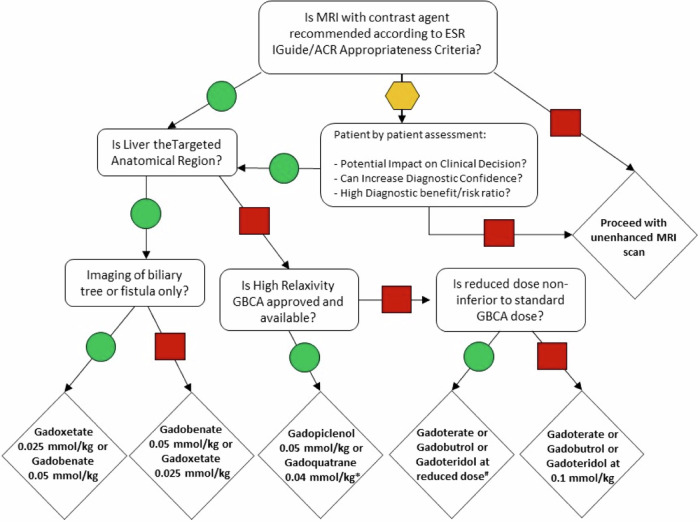
Decision Flow chart for indication to use GBCA, choice of GBCA type, and dosing options when receiving an imaging referral for a contrast-enhanced MRI scan. The arrow with a green circle means “Yes”; the arrow with a red square means “No”; the arrow with a yellow hexagon means “Uncertain”. Uncertainty depends on the clinical scenario, patient history, and additional clinical features that are not contemplated in the appropriateness criteria. *Note that gadopiclenol is approved while gadoquatrane is an investigational product in phase III clinical trials. ^#^Reduced doses must be applied according to available data as suggested by current and updated evidence-based medicine. ESR, European Society of Radiology; ACR, American College of Radiology

Dose optimization is recommended, as administering a lower-than-standard dose can still provide diagnostic information that is equally effective; however, it must be acknowledged that disease processes, and potentially MRI settings, influence contrast enhancement. Beyond this effort, it is common sense that, before considering dosage options, the radiologist must assess clinical indication, feasibility, appropriateness, and necessity of injecting GBCA to solve a clinical question. This includes a risk-benefit analysis for each patient and according to updated guidelines and scientific evidence) [2, 26] (Fig. 1).

In the oncology setting, the need for structured reporting for a standardized approach to scan protocols and patient management, has opened the discussion on the role of GBCA during the diagnostic workup at first diagnosis of cancer. The most recent overview on this topic has highlighted the key role of enhanced MRI in most MRI-based structured reporting and data systems (RADS) [6].

It must be acknowledged that radiologist expertise and confidence play a key role in the choice of GBCA injection during an MRI scan, independent of the clinical indication. There is wide agreement that in all pathology and organ settings, GBCA-enhanced MRI is crucial in the ability to detect space-occupying lesions through an increase of contrast-to-noise ratio, to characterize pathological tissue through enhancement patterns, to quantify tissue perfusion, to show treatment response and sequelae through intraindividual enhancement pattern changes, therefore influencing the clinical decision.

However, despite recommendations being available for most common diseases, the level of scientific evidence remains moderate and, in general, it is common practice to prescribe an MRI scan with contrast (rather than without contrast) as it increases confidence and reduces the risk of false negatives in radiological reports.

Further research is necessary to increase appropriateness in the use of GBCA in different clinical contexts to understand the real improvement in patient care by adding contrast-enhanced imaging. Since 2014, the ESR has been working with the American College of Radiology (ACR) to develop referral guidelines for Europe, based on the ACR Appropriateness Criteria [27]. The ESMRMB-GREC working group keeps seeking the collaboration of the other ESR subspecialty societies to develop educational resources for radiologists in this regard.

## Lowering to zero-dose using AI: Gain or pain?

GBCA standard dose is 0.1 mmol/kg of body weight for most applications of gadobutrol, gadoterate meglumine, gadoteridol; 0.05 mmol/kg of body weight for liver imaging using gadobenate dimeglumine, and 0.025 mmol/kg of body weight for liver imaging using gadoxetate disodium. Dose optimization is recommended and administration of lower than standard dose has been shown to provide non-inferior diagnostic information, in specific contexts [28].

During the last 5 years, the rapid evolution of artificial intelligence-based applications in radiology has not only opened an intense debate but also a research trend toward the development of IT solutions with the scope of lowering the GBCA dose down to zero while maintaining diagnostic accuracy [29]. Indeed, a new frontier in MRI is foreseen if the same amount of MRI information can be obtained without injecting GBCAs (virtual enhancement) or with a lower dose (augmented enhancement), at the same time gaining in scanner time efficiency, lower costs, and better patient and staff comfort.

The GBCA zero-dose option (virtual enhancement), although extremely fascinating, remains difficult to accept for diagnostic use as numerous factors influence contrast enhancement. In fact, MRI signal enhancement is generated by the dipolar interactions between water protons and electron spins at the GBCA metallic center. Therefore, the amount of water protons in tissues and the amount and vicinity of gadolinium ions to water protons have a profound impact on contrast enhancement. This is the reason why tissue distribution (extracellular for all of those available and intracellular for those that are partially excreted via the hepato-biliary system) of GBCA, the microvessel tissue density, and endothelial permeability in healthy and pathological states, affect the extent of contrast enhancement. In addition, the tissue distribution of GBCA is a time-dependent phenomenon; in particular, tissue perfusion measurements are strongly dependent on haemodynamics, and late gadolinium enhancement depends on tissue compartmentalization [30, 31]. Last but not least the spatial resolution and signal-to-noise ratio vary with different MR hardware/software systems as an additional challenge of artificially reconstructing enhanced images from unenhanced raw data, especially at boundary locations and with small enhancing lesions [29].

In summary, virtual contrast-enhancement with zero-dose of GBCA, despite its feasibility through the use of deep learning algorithms, is unlikely to reflect pharmacokinetic (i.e., tissue distribution and concentration) events in the single patient and therefore it is expected to be clinically unreliable.

The low-dose GBCA option, on the other side, aims at obtaining virtual full-dose images through signal and/or contrast augmentation of real images obtained with the injection of low GBCA doses, down to 10% of the standard dose [29]. This approach not only is feasible but is a measure of tissue distribution and concentration of GBCA [32] and at least partially reflects all different factors mentioned above, therefore holding more promise than the zero-dose option.

## Next-gen MRI contrast agents: something’s brewing

The European Medicines Agency recently released authorization for gadopiclenol to improve the quality of the resulting MRI scan compared to an unenhanced scan [33]. Gadopiclenol is a new extracellular GBCA with a comparable safety profile and stability but higher relaxivity than other approved macrocyclic GBCA. Based on its hydration number of 2, two water nuclei interact with the gadopiclenol-caged gadolinium ion, thus reaching a 2–3-fold increase of relaxivity and similar contrast enhancement at a 50% lower dose than that approved for standard GBCA. The PICTURE and PROMISE randomized clinical trials have indeed shown that 0.05 mmol/kg gadopiclenol is not inferior to 0.1 mmol/kg gadobutrol for MRI of the CNS and body, and achieves similar clinical efficacy [34, 35].

Gadoquatrane is the other high-relaxivity macrocyclic GBCA that is currently undergoing phase 3 clinical trials [36]. This new GBCA has been designed as a tetramer with a 2- to 3-fold increase in relaxivity per molecule and a comparable diagnostic efficacy at a 40% lower dose than that approved for standard GBCA [36]. The multicenter, randomized, prospective, double-blind, cross-over studies (Quanti OBR and Quanti CNS) are currently running on the efficacy and safety in adults [37, 38]. Also, a multicenter, prospective, open-label study (quanti pediatric) is currently recruiting to evaluate pharmacokinetics and safety in children below 18 years [39].

Manganese-based contrast agents (MBCAs) represent an alternative to GBCAs. Such new compounds take advantage of the paramagnetic properties of manganese and are currently under clinical development. The oral manganese chloride tetrahydrate underwent the SPARKLE phase 3 clinical trial to evaluate its safety and diagnostic efficacy for imaging of focal liver lesions in patients with severe renal impairment [40]. Up to date, it is in Europe at the stage of investigational medicinal product and not approved. Another MBCA designed for intravenous injection and to be compared with extracellular GBCA is RVP-001 (Mn-PyC3A) which is undergoing the phase 2 trial to evaluate safety and efficacy and to identify an appropriate dose to detect brain lesions in adults [41]. Although not new, clinical trials on the use of ultra-small superparamagnetic iron oxide contrast agents (ferumoxytol and ferumoxtran-10) in the study of central nervous system tumors, prostate cancer, and lymph node metastases are currently recruiting [42–44]. The above-listed compounds can be considered game-changers in the world of contrast-enhanced MRI because they will reduce the amount of gadolinium administered to patients and therefore emitted into the environment. It is expected that not only the completion of mentioned phase 2 and phase 3 trials, but also post-marketing surveillance trials will need to confirm their safety and diagnostic efficacy.

## Summary statement

GBCAs are currently used in up to 50% of all MRI exams in Europe. Various concerns have been raised for the safety of patients and the environmental impact, guiding the research (preclinical and clinical) toward the reduction of GBCA in MRI. However, the properties of extracellular and liver-specific GBCA are unique and add substantial information to unenhanced images for guiding patient care. The recommendations on clinical appropriateness of enhanced MRI, the effort on testing lower doses of standard GBCA to reach comparable diagnostic efficacy, and the development of AI solutions to augment contrast-enhancement and next-generation MRI contrast agents will reduce the overall need for gadolinium-related resources, therefore increasing allocative and technical efficiency of MRI.

## Patient summary

Current MRI contrast agents have an excellent safety profile. However, in some clinical contexts, the intravenous injection of a contrast agent does not add any information and could be avoided. The use of GBCAs must be justified in terms of benefit/risk ratio and optimized to the lowest dose achievable without losing diagnostic information. Next-generation contrast agents show promise to obtain comparable diagnostic efficacy and safety with lower doses.

## Abbreviations

ESUR-CMSC: Contrast Media Safety Committee of the European Society of Urogenital Radiology
GBCAs: Gadolinium-based contrast agents
GREC: Gadolinium Research and Education Committee
MBCA: Manganese-based contrast agents
